# Feasibility of an online training and support program for dementia carers: results from a mixed-methods pilot randomized controlled trial

**DOI:** 10.1186/s12877-022-02831-z

**Published:** 2022-03-01

**Authors:** Soraia Teles, Ana Ferreira, Constança Paúl

**Affiliations:** 1 Faculty of Medicine, University of Porto (FMUP), Alameda Prof. Hernâni Monteiro, 4200-319 Porto, Portugal; 2 Center for Health Technology and Services Research (CINTESIS), Rua Dr. Plácido da Costa, 4200-450 Porto, Portugal; 3 School of Medicine and Biomedical Sciences University of Porto (ICBAS-UP), Rua de Jorge Viterbo Ferreira, 228, 4050-313 Porto, Portugal

**Keywords:** Dementia, Informal Carers, Online program, Knowledge and skills training, Pilot study, Portugal

## Abstract

**Background:**

iSupport is an online program developed by the World Health Organization to provide education, skills training, and social support to informal carers of persons with dementia. This pilot study examines the feasibility of the protocol for a main effectiveness trial of iSupport-Portugal and explores how the intervention and control arms compare over time on well-being outcomes.

**Methods:**

A mixed-methods experimental parallel between-group design with two arms is followed. Participants were recruited nationwide, by referral or advertising, through the National Alzheimer’s Association. Inclusion criteria are being Portuguese adults, providing e-consent, providing unpaid care to someone with dementia for at least 6 months, experiencing relevant scores on burden (≥ 21 on ZBI) or depression or anxiety (≥ 8 on HADS), and using webpages autonomously. Participants were consecutively randomized to receive iSupport-Portugal or an education-only e-book and were not blinded to group assignment. Data were collected online with self-administered instruments, at baseline, 3 and 6 months after. Outcomes comprise caregiver burden, depression, anxiety, QoL, positive aspects of caregiving, and self-efficacy. Generalized estimating equations were used to estimate group, time, and group-by-time effects. Intervention engagement data were extracted from iSupport’s platform. Semi-structured interviews were conducted.

**Results:**

Forty-two participants were allocated to the intervention (*N* = 21) and control (*N* = 21) arms. Participation (78.1%) and retention rates (73.8%) were fair. More carers in the control arm completed the study (*N* = 20, 95.2%) than in the intervention arm (*N* = 11; 52.4%) (*χ*^2^ = 9.98, *p* = .002). Non-completers were younger, spent less time caring, and scored higher on anxiety. Among carers in the intervention arm, the average attendance rate was of 53.7%. At post-test 38.9% of participants still used iSupport; the remainder participants interrupted use within 2 weeks (Mdn). For per-protocol analyses, significant group-by-time interaction effects favouring the intervention were found for anxiety (Wald χ2 = 6.17, *p* = .046) and for environmental QoL (Wald *χ*^2^ = 7.06, *p* = .029). Those effects were not observed in intention-to-treat analyses adjusted for age. Interviewees from the intervention arm (*N* = 12) reported positive results of iSupport on knowledge and on experiencing positive feelings. No adverse effects were reported.

**Conclusions:**

This study provides information for a forthcoming full-scale effectiveness trial, as on the acceptability and potential results of iSupport-Portugal. iSupport is suggested as a relevant resource for Portuguese carers.

**Trial registration:**

ClinicalTrials.gov, NCT04104568. 26/09/2019.

**Supplementary Information:**

The online version contains supplementary material available at 10.1186/s12877-022-02831-z.

## Introduction

Worldwide, 50 million people are estimated to live with dementia [1]. Among older adults, dementia is the leading chronic condition causing disability and dependency [2]. Most people with dementia live at home [84% globally [3]] and rely on support from family members, parallel or not with formal services. Informal carers may provide unpaid and ongoing assistance with basic or instrumental activities of daily living or organize care delivery by others [4].

Carers of persons with dementia often care for several years, while being exposed to stressors, including financial problems, time constraints, and care management issues [2]. While the impact of caregiving is highly individualized, and both positive and negative aspects may coexist [5], research has demonstrated that dementia carers experience strain at practical, physical, and psychological levels [6]. When compared to the general population, dementia carers are at a greater risk of developing depression and anxiety, hypertension, digestive, and breathing problems [4, 7, 8]. Carers’ psychosocial variables, including stress, low sleep quality, mental disorders, or perceived inability to provide care, were related with harmful behaviours towards the care recipients, and predicted their institutionalization [9–11].

To prevent or minimize deleterious outcomes for both the carers and the care recipients, psychosocial interventions are instrumental. Internet interventions have been explored as a mean to expand training and support to dementia carers, either as a complement or in alternative to usual care. When self-guided or minimally supported by professionals, these interventions are easily scalable at a relatively low marginal cost per additional user [12]. Internet interventions are accessible and, when self-guided/or not scheduled, are self-paced and available around-the-clock. Research on the acceptability of internet interventions is encouraging, with carers most frequently valuing its accessibility, convenience, and the opportunity to avoid stigmatized professional help [13, 14]. Beneficial effects were shown, more robustly, on depression [12, 14–18], but also on anxiety [12, 15, 16, 19], stress [12, 16, 18, 19], burden [12, 17, 18], and self-efficacy [16, 17, 20]. In recent reviews, the effects of internet and face-to-face interventions on carers’ emotional well-being were found to be equivalent [20, 21]. In presenting a European consensus on outcome measures for psychosocial intervention research in dementia care, Moniz-Cook and colleagues [22] proposed the assessment of mood, burden and quality of life in family carers, while stressing the need of measures and of measuring positive aspects of care.

‘iSupport’ was developed by the World Health Organization to provide online and self-guided education, skills training, and social support to carers experiencing stress, burden, and mild/moderate symptoms of depression or anxiety [23]. A positive and rewarding involvement with the person with dementia throughout care provision (positive aspects of care) is promoted. The program is based on the model of Kitwood, proposing that the personhood of someone diagnosed with dementia is key, thus behaviour must be understood not only as a reflexion of brain functioning but also as a product of personality, life history, health status, coping strategies, and social, as well as physical environment [24]. Thus, care is thought of as interaction in accordance with individuals’ needs, abilities, and personality. iSupport uses problem-solving and cognitive behavioural therapy techniques, including psychoeducation, behavioural activation, cognitive reframing, relaxation, communication training, and antecedent-behaviour-consequence analysis [23].

iSupport was developed as a generic version requiring cultural adaptation to each implementation setting. In accounting for identified needs, iSupport was culturally adapted to Portugal [25]. The country is positioned above the OECD average on the prevalence of dementia with 21 cases per 1000 inhabitants [26]. Most Portuguese carers care on a daily basis (8.2%), and in a higher percentage than the OECD average [26]. Compared to other 15 European countries, Portugal has the highest rate of co-residential care (12.4%), which is a more intensive type of care [27]. Portuguese dementia carers have reported high psychological needs [28, 29], a negative appraisal of support services availability [29, 30], and contextual barriers to access face-to-face interventions [29]. In examining the potentials of internet interventions to bridge such gaps, attitudes of digitally literate Portuguese carers towards online interventions were found to be positive [31], and a good match was observed among carers’ training needs and iSupport features [32].

RCTs aimed at determining the effectiveness of iSupport were completed in India [33] and the Netherlands [34]. Results published for the former^1^ show improvements in the iSupport group on person-centered attitude towards persons with dementia, but not in other outcomes [35]. The trial had high attrition, hampering more robust conclusions [35].

This paper describes a mixed-methods pilot randomized controlled trial of iSupport-Portugal. In Portugal, there is no similar research that could set a reference for trial parameters, such as referral, eligibility, participation, or dropout rates. This pilot is a small-scale version of an upcoming full-scale effectiveness study. Components and processes planned for the latter [36], including the randomisation of participants, are implemented in this pilot and examined for its feasibility. A qualitative research component is added to provide information about the processes of using iSupport and of participating in the pilot study. This paper also explores how the intervention and control arms compare over time on the outcome measures of burden, depression and anxiety, positive aspects of caregiving, self-efficacy, and quality of life.

## Materials and methods

### Study design

A mixed-methods randomized controlled trial with two arms (iSupport-Portugal vs. education-only e-book) was followed. A factual analysis based on qualitative data allows to address the processes of how and why an intervention may work/not work. The study is single blinded as participants are aware of the intervention received. Assessments were taken at baseline (T_0_), 3 months (post-test/T_1_) and 6 months (follow-up/T_2_) after baseline using self-administered instruments, filled out online, with no interference of researchers.

Attrition prevention measures included: 1) sending out analogous weekly email reminders to participants in both arms, to either use iSupport or check the e-book. Reminders were sent from baseline to post-test; 2) contacting, a week after allocation, the participants in iSupport’s arm not yet registered into the program, to check for technical difficulties; and 3) sending out up to two email reminders to fill in post-test and follow-up assessments.

The study protocol is published elsewhere [36]. Minor changes to the protocol were needed and are described later in this section. Ethical approval was granted by the Ethics Committee for Health of the São João University Hospital Center/Faculty of Medicine, University of Porto (Ref. 208/18). The study adheres to CONSORT guidelines; the Additional file 1 provides the CONSORT checklist.

### Intervention condition: iSupport-Portugal

**Fig. 1:**
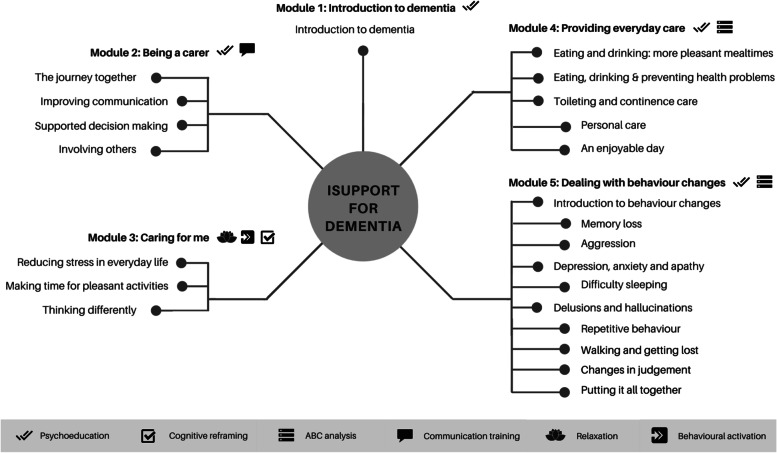
iSupport modules, lessons, and psychological techniques. Adapted from [38]. Lessons names vary slightly in the European-Portuguese version

iSupport-Portugal is the European-Portuguese version of WHO’s iSupport for dementia. The process of culturally adapting iSupport is described elsewhere [25]. A five-step methodological approach was used to culturally adapt iSupport to Portugal which comprised: 1) needs assessment, 2) content translation by an authorized translator and technical accuracy check of the translation by health and social support professionals, 3) cultural adaptation for semantic and conceptual equivalence of expressions, habits, traditions, local resources and practices), 4) independent appraisal of contents by an expert panel, and 5) fidelity check by the authors of the program (WHO) [25]. The usability of iSupport-Portugal was tested with carers and health/social support professionals [37]. The online knowledge and skills training program can be accessed anytime/anywhere by registered users via a web interface. The program comprises 5 modules and 23 lessons which are mostly text-based. Figure 1 details the topics in each lesson and the psychological techniques employed. iSupport is self-guided, offers full flexibility regarding the intervention schedule, and carers may decide on their own lessons plan. To promote engagement, iSupport includes personalization features (e.g., text contents are personalized with care recipient’s sociodemographic data), is populated with caregiving scenarios, and includes interactive skills-training exercises. A mood self-assessment tool allows carers to self-monitor their mood status over time.

### Comparison condition: education-only e-book

The minimal education-only intervention offered to the control arm consists on the European-Portuguese version of the ‘Care Manual’ [39]. The e-book contains comprehensive information on relevant topics for dementia and caregiving, and its thematic contents overlap with issues approached in iSupport. Participants in the control group could access iSupport after study completion.

### Participants, randomization, and blinding

Carers were recruited nationwide (Portugal) over 8 weeks (from March 2020). Whitehead and colleagues [40] recommend that for a main trial designed with 80% power, two-sided 5% significance, and accounting for medium standardised effect sizes (the assumptions for the main iSupport trial [36]) the pilot sample sizes per arm must be of at least 10. Two recruitment pathways were possible: 1) by referral from health/social support professionals from the National Alzheimer’s Association; 2) by volunteering to participate in response to advertising on social media pages of the same association.

Participants were consecutively selected as they met the eligibility criteria: 1) being Portuguese adults (≥18 years), 2) providing non-paid care for at least 6 months, 3) to a person with a formal diagnosis of dementia, 4) experiencing a clinically relevant level of subjective burden [score ≥ 21 on the Zarit Burden Interview, Portuguese version [41]], or depression or anxiety symptoms [score ≥ 8 in respective subscales of the Hospital Anxiety and Depression Scale, Portuguese version [42]],^2^ 5) being capable of using the internet autonomously, and 6) having provided e-consent to participate in the study. Participants unable to understand written Portuguese, not having access to an internet connected device at least twice a week, and/or reporting that the person with dementia is in institutional care, would be excluded from the study, but given with the opportunity to access iSupport.

Referred participants authorized the referring professional to share their email address with the research team and were contacted. Volunteers approached the research team either by email or telephone. In both referral-based and advertising-based recruitment, complete study information was sent out by email and participants were asked to reply in confirmation or denial of their willingness to participate. Consenting carers received a link to the fill-in form aimed at determining the compliance with eligibility criteria, and at assessing the participants at baseline. Forms went through quality control to identify any inconsistent answers potentially affecting participant’s eligibility and participants were contacted in such cases. Participants recruited by advertising were phone-interviewed about the diagnosis history of the person in care as, in contrast to the referred carers, there was no confirmation of the dementia diagnosis by professionals.

After filling the online form, eligible participants were randomized (permuted block) to the intervention or control arms. The participants’ order was defined by the form submission date/time stamp; the researchers had no control over the assignment. Participants were notified about the allocated group and provided with the respective information/materials: access code and instructions to access iSupport, or the e-book. All participants in the intervention arm were invited to participate in a one-to-one online interview, including dropouts.

### Variables and measures

At baseline, data were collected on: 1) sociodemographic characteristics of carers, 2) sociodemographic characteristics of care recipients, 3) care recipients’ health, diagnosis-related information and degree of dependence (subjectively evaluated by the carer), 4) caregiving context, 5) general use of the internet by carers, and 6) attitudes towards online psychoeducational programs, measured by the Online Psychoeducational Interventions-Brief Attitudes Scale [31]. Variables are specified on Table 2.

To follow-up on relevant changes over time, some variables were reassessed at post-test and follow-up, including: continuation/discontinuation of care, carer occupational status, care recipient degree of dependence (perceived by the carer), number of hours spent caring, support for caregiving, cohabitation, and attitudes towards online psychoeducational interventions. Carers who discontinued care were asked about the motives and time elapsed since discontinuation.

#### Feasibility and engagement measures

Feasibility measures comprise: 1) participation rate: participants filling the screening form / participants referred or volunteering to participate × 100%; 2) non-eligibility rate: participants not eligible / participants filling the screening form × 100%; and 3) study dropout (post-test and follow-up): participants not completing the post-test or follow-up / participants enrolled × 100%.

Engagement measures are examined for participants allocated to the intervention arm (*N* = 21) and based on data extracted from iSupport’s web platform: 1) non-use attrition: participants not registered into iSupport / participants allocated to the intervention group × 100%; 2) intervention dropout attrition: participants visiting less than five iSupport’s lessons / participants allocated to the intervention group × 100%; 3) usage rate at post-test: participants who used iSupport at post-test / participants registered into iSupport × 100%; 4) time until discontinuation: weeks elapsed since the first and last visits to iSupport until post-test; the last visit is defined as the last entry followed by an inactivity of at least 4 weeks; 5) number of logins into iSupport from baseline to post-test; 6) number of lessons visited until post-test; 7) attendance rate: lessons visited / total number of lessons × 100%; 8) percentage of registered participants who visited each iSupport module; and 9) percentage of registered participants who used the mood rating function of iSupport. As iSupport includes a printout function, usage data was inspected for ‘binge printing’ followed by inactivity, possibly suggesting offline use.

#### Outcomes

Study outcomes were assessed through self-report instruments validated to Portugal and comprised: 1) perceived caregiver burden - Zarit Burden Interview total score [41], 2) symptoms of depression and 3) of anxiety - total scores of the respective subscales of the Hospital Anxiety and Depression Scale [42], 4) positive role appraisals - total score on the Positive Aspects of Caregiving [43], 4) general self-efficacy - total score of the Generalized Self-efficacy Scale [44], and 5) subjective quality of life (overall, physical, psychological, social relationships, environment) - WHOQOL-BREF raw scores for each domain [45]. To explore how the intervention and control arms compare over time on outcome measures, only total scores rather than cut-offs were considered. Cut-off scores for HADS-anxiety and HADS-depression subscales are used to describe the sample (see Results) as normal (0–7 points), borderline cases (8–10 points), or abnormal cases (11–21 points) [42]. Outcomes were measured at baseline, 3 and 6 months after.

#### Qualitative data

An interview guide was developed to gather data on issues detailed on Table 1. The influence of the COVID-19 pandemic on this study was examined, as the study start coincided with the lockdown in Portugal. A semi-structured format was adopted. Audio recorded interviews were carried after the follow-up measurements, from November 2020 to January 2021.

**Table 1:** Semi-structured interview: issues covered

**Motivations to participate in the study** **Advantages/disadvantages of online training and support** • Advantages, if any, of getting online training and support • Advantages/disadvantages of online interventions as compared to face-to-face	**Perceived results of iSupport** • Perceived positive or negative results of iSupport • Perceived persistence of positive results (if any) over time • (If positive results are not perceived) features that the program should have to produce positive results
**Usage of iSupport** • Continuation/discontinuation of use • Motives to keep using the program/to have discontinued • Satisfaction with the frequency of using iSupport • Obstacles to use/a more frequent use of iSupport • Description of program usage - Choosing lessons - Timing/schedule and place - Devices	**Satisfaction with contents, design, and functionalities** • Satisfaction with: - Interface appearance and easiness of use - Themes - Language - Content presentation - Self-guidance • Missing features
**Program endorsement and user profile** • Willingness to recommend iSupport to other carers • Beliefs on iSupport usefulness for other carers • Perceived profile of carers who would use iSupport	**Concomitant use of psychosocial services** • Use of psychosocial interventions during the study • Perceived positive/negative results of such interventions • (if not used) Reasons for not having used other services
**COVID-19 pandemic** • Influence (if any) of the pandemic on using iSupport • Loss/suspension of other psychosocial supports • Motivation to keep using iSupport after recovering other forms of support (if applicable)	**Study procedures, inconvenience, or harm** • Perceived inconvenient or arms (of the intervention and study procedures, including reasons for not filling out all assessment waves) • Influence (if any) of weekly reminders on program usage

### Data analysis

Descriptive statistics are used to characterize study participants, as well as feasibility and engagement measures. Absolute and relative frequencies, central tendency (mean, median) and dispersion (range, interquartile range, standard deviation) measures were used as appropriate. The study completers of each arm were compared at baseline, and study completers were also compared with non-completers at baseline across all measured variables (e.g., sociodemographic, caregiving context data) and outcome measures. Mann-Whitney U Test, and chi-square tests were used as appropriate. The Friedman test was used to assess changes over time in participants’ attitudes towards online psychoeducational interventions.

The analyses were performed first according to a per-protocol approach by excluding the carers who did not fill all three assessment waves (study non-completer). None of the participants visiting less than five lessons of iSupport (defined as the per-protocol criterion for intervention adherence) have completed the assessment waves, thus non-completers are also intervention dropouts. In addition to the per-protocol approach, intention-to-treat (ITT) analyses were carried out. Multiple imputation with chained equation (MICE) with 20 imputations/datasets was used to address missing values. Generalized estimating equations (GEE) analyses were used to estimate between and within-group effects on each outcome. Because there were no significant group differences at baseline among study completers (Table 2) no adjustments were made for sociodemographic, caregiving context, or any other data collected about carers and care recipients for per-protocol analyses. For ITT analyses, relevant age differences at baseline between participants randomised to each arm (i.e., Standardized difference > 0.5 and *r* > 0.3 with outcome variables) required adjustments for this variable. The quasi-likelihood information criterion (QIC) was used to select the best correlation structure and best fitting model in GEE analysis; the Gamma distribution was selected, for which the lowest QIC was found for all outcomes. All *p* values are two-sided with a significance level of 0.05. The required sample size for a full-scale study would be of *N* = 184 [36]; thus within-between group comparisons performed with pilot data are exploratory.

For interviews data, a thematic content analysis was performed after transcribing the recordings. The analysis (using NVivo 11 software) followed a horizontal scheme, and the categories were defined in a data-driven approach.

### Adjustments to the study protocol

Adjustments to the study protocol [36] included recruiting participants by advertising in addition to a referral-based recruitment, as the last was compromised by the suspension of psychosocial services during the lockdown. The recruitment method was accounted in analysing feasibility data.

System inclusion/exclusion of participants according to eligibility criteria was foreseen; however, due to technical limitations, the criteria were verified manually by the researchers.

No changes were made on outcomes; however, the version of the Zarit Burden Interview distributed by MAPI Research Trust (which holds the distribution rights for ZBI) [41] was used in alternative to Gonçalves-Pereira’s version [46]. There are no major differences between the two versions, but MAPI issued a high-quality European Portuguese translation of the instrument using linguistic validation methods. A measure of attitudes towards online psychoeducational interventions [31] was included.

## Results

**Fig. 2:**
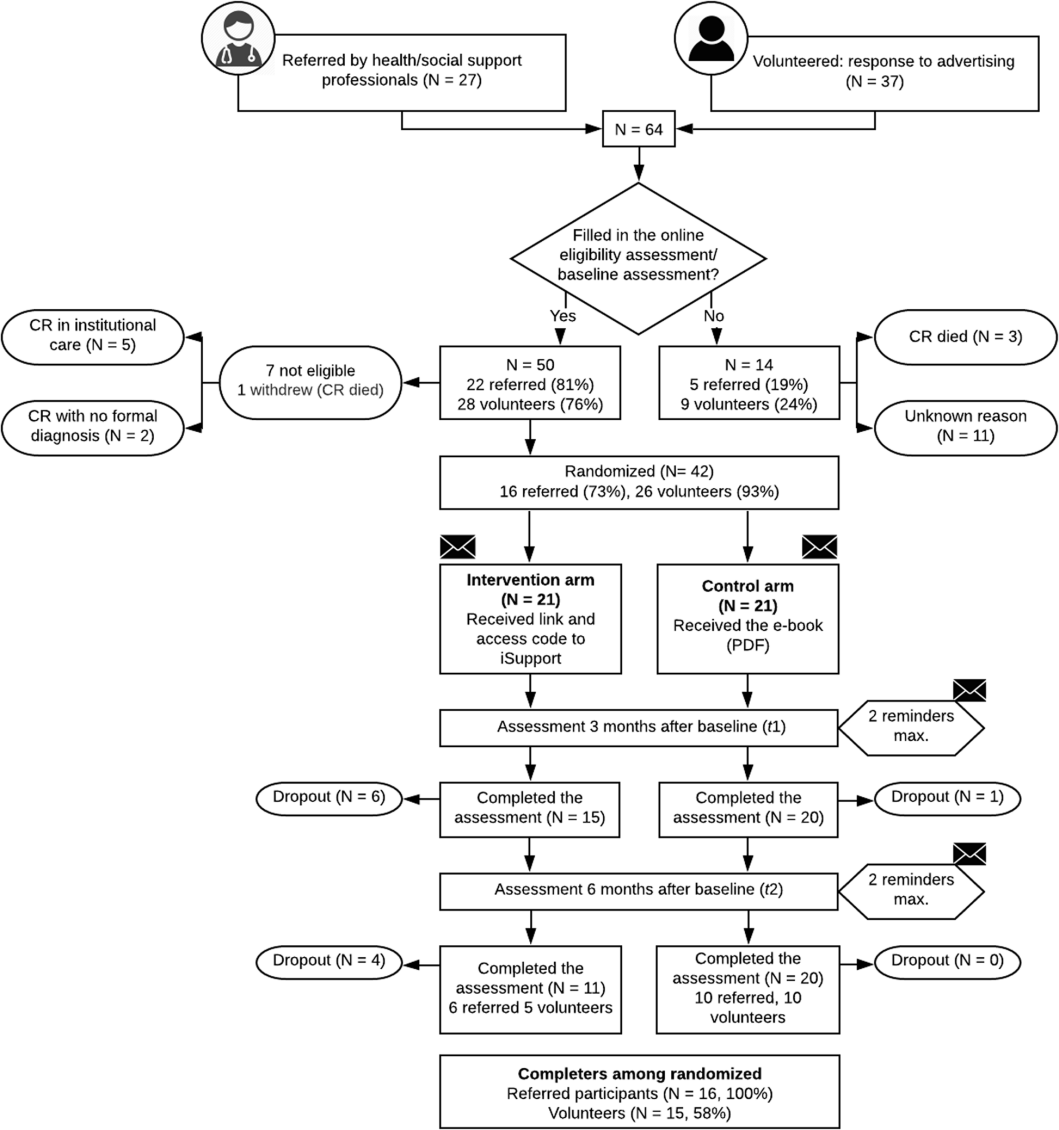
Diagram for the pilot study. CR – Care recipient. N – number of participants

After exclusion of non-eligible participants (Fig. 2), 42 carers were randomized to either iSupport (*N* = 21) or the education-only (*N* = 21) condition. All excluded participants (14%) failed to comply with context of care criteria. Thirty-one participants completed all assessment waves, 11 in iSupport arm and 20 in the control arm (study completers). The completion rate in the two groups is significantly different (*χ*^2^_(1, *N* = 42)_ = 9.98, *p* = .002).

### Characterization of study participants at baseline

Most carers who were randomized (*N* = 42) are female (78.6%), middle-aged (M 53.6 years, SD 13), and highly educated (M 15.5 years of schooling, SD 4.1). Most are offspring carers (73.8%; two grandchildren; the remaining are adult children), provide intensive (Mdn 29 h./week, IQR 43, range 5–168), and long-term care (Mdn 3, IQR 3.25, range 6 months-20 years). Most receive support for caregiving (71.4%), especially from other informal carers (40.5%). The internet is used daily by 90.5%. Smartphones (78.6%) and laptops (73.8%) are the most used devices. Carers presented good *à priori* attitudes towards online interventions: 78.6% scored 20 or more on OPI-BAS, the proposed cut-off to discern sympathizers from non-sympathizers of online interventions [31].

Most care recipients are female (76.2%), with 76.3 years on average (SD 9.9). Most are diagnosed with Alzheimer’s disease (69%) and live in their own home (76.2%; others live in relatives’ home).

At baseline, all 42 randomized carers presented relevant levels of burden (M 37.3, SD 10.8). For anxiety symptoms (M 9.7, SD 4), 12 carers (28.6%) would be classified as normal, 13 (31%) as borderline cases, and 17 (40.5%) as abnormal cases. For depression (M 7, SD 4.7), 23 carers (54.8%) are not a case, 9 show borderline values (21.4%) and 10 (23.8%) abnormal values.

Table 2 disaggregates the information by group. Study completers (responding to all three assessment waves) are, at baseline, statistically different from non-completers. Younger carers (U = 96.5, *p* = .034), those spending less time caring (U = 95, *p* = .031), and those scoring higher on anxiety (U = 77, *p* = .007) dropped out more. Study completers in both arms are not statistically different with regards to variables used to characterize them, or to outcome measures.

**Table 2:** Characteristics of participants on the intervention and control arms at baseline: randomized sample and study completers

	Randomized sample		Study completers (SC)			SC (***N*** = 31) vs. NC (***N*** = 11)
	iSupport (*N* = 21)	Control (*N* = 21)	iSupport (*N* = 11)	Control (*N* = 20)	***p***	***p***
**Socio-demographic data: caregiver**						
Age (years), M (SD)	49 (12.1)	58.1 (12.5)	52.2 (10.9)	58.8 (12.4)	.166^a^	**.034**^a^
Gender, Female, n (%)	17 (81.0)	16 (76.2)	9 (81.8)	15 (75.0)	1^b^	1^b^
Years of schooling, M (SD)	15.8 (4.2)	15.2 (4.2)	15.6 (5.0)	15.1 (4.2)	.520^a^	.405^a^
Marital status, Partnered, n (%)	12 (57.1)	14 (66.7)	7 (63.6)	14 (70.0)	1^b^	.281^b^
Occupational status, Employed, n (%)	14 (66.7)	13 (61.9)	7 (63.6)	12 (60.0)	1^b^	.717^b^
**Socio-demographic data: care recipient**						
Age (years), M (SD)	73.8 (10.7)	78.9 (8.4)	75.3 (9.1)	79.2 (8.6)	.215^a^	.169^a^
Gender, Female, n (%)	17 (81.0)	15 (71.4)	9 (81.8)	15 (75.0)	1^b^	1^b^
**Diagnosis & health-related: care recipient**						
Type of dementia					.698^b^	.453^b^
Alzheimer’s disease, n (%)	16 (76.2)	13 (61.9)	8 (72.7)	12 (60.0)		
Other/unknown, n (%)	5 (23.8)	8 (38.1)	3 (27.3)	8 (40.0)		
Time since diagnosis (years), Mdn (IQR)	4 (5)	4 (4.5)	2 (3)	3.5 (4.8)	.134^a^	.349^a^
Dependence level					.707^b^	.484^b^
Mild/moderate, n (%)	10 (47.6)	11 (52.4)	7 (63.6)	10 (50.0)		
Total/severe, n (%)	11 (52.4)	10 (47.6)	4 (36.4)	10 (50.0)		
**Caregiving context**						
Caregiving duration (years), Mdn (IQR)	3 (3)	3 (5.5)	3 (2)	3 (6.3)	.786^a^	.517^a^
Hours caring (per week), Mdn (IQR)	30 (46)	28 (71)	36 (36)	29 (73.5)	.679^a^	**.031**^a^
Support for caregiving, Yes, n (%)	15 (71.4)	15 (71.4)	7 (63.6)	14 (70.0)	1^b^	.464^b^
Relationship with the care recipient					1^b^	.234^b^
Offspring, n (%)	17 (81.0)	14 (66.7)	8 (72.7)	13 (65.0)		
Spouses, n (%)	4 (19.0)	7 (33.3)	3 (27.3)	7 (35.0)		
Cohabitation, Yes, n (%)	14 (66.7)	13 (61.9)	9 (81.8)	12 (60.0)	.262^b^	.481^b^
**Internet use & attitudes towards OPIs**						
Internet use frequency, Mdn (IQR)	1 (0)	1 (0)	1 (0)	1 (0)	.644^a^	.216^a^
Attitudes towards OPIs (OPI-BAS), M (SD)	22.3 (2.6)	21.3 (2.3)	22.5 (2.6)	21.5 (2.3)	.249^a^	.524^a^
**Outcome measures at baseline**, M (SD)						
Caregiver burden (ZBI)	37.7 (11.3)	37 (10.9)	37.8 (13.1)	37.1 (11.2)	.901^a^	.897^a^
Anxiety symptoms (HADS-A)	11.2 (4.0)	8.3 (3.6)	10.1 (4.6)	8 (3.4)	.165^a^	**.007**^a^
Depression symptoms (HADS-D)	8 (5.2)	6 (3.9)	6.6 (5.2)	6.1 (4.0)	.983^a^	.105^a^
Positive aspects of caregiving (PAC)	36.2 (9.3)	39.6 (8.2)	35.8 (6.1)	39.6 (8.4)	.159^a^	.920^a^
General Self-efficacy (GSE)	29.8 (5.4)	31.2 (4.6)	28.5 (4.9)	31.3 (4.7)	.190^a^	.818^a^
Quality of Life (WHOQOL-BREF)						
Physical	26 (4.1)	26.8 (4.3)	26.5 (4.4)	27 (4.4)	.739^a^	.154^a^
Psychological	21.6 (4.5)	22.8 (3.7)	22.7 (4.6)	22.9 (3.8)	.885^a^	.108^a^
Social relationships	9.7 (2.9)	10.3 (2.7)	10.4 (2.8)	10.1 (2.6)	.492^a^	.428^a^
Environment	29 (4.5)	29.5 (4.2)	29.3 (4.0)	29.3 (4.2)	.967^a^	.730^a^
General	7.2 (1.3)	6.9 (1.6)	7.6 (1.3)	6.8 (1.5)	.131^a^	.883^a^

*Abbreviations*: *SC* study completers, *NC* non-completers, *N* number of participants, *M* mean, *Mdn* median, *SD* standard deviation, *IQR* interquartile range, *OPIs* online psychoeducational interventions, *OPI-BAS* Online Psychoeducational interventions – Brief Attitudes Scale, *ZBI* Zarit Burden Interview, *HADS-A* Hospital Anxiety and Depression Scale (anxiety subscale), *HADS-D* Hospital Anxiety and Depression Scale (depression subscale), *PAC* Positive Aspects of Caregiving, *GSE* Generalized Self-efficacy Scale

^a^ Tested with Mann-Whitney U Test; ^b^ Chi-square tests (Yates’ Correction for Continuity for 2 × 2 tables or Fisher’s Exact Test). Values in bold represent the statistically significant differences at *p* < .05

### Changes in the caregiving context and on attitudes over time

Among study completers (*N* = 31), 4 (12.9%) discontinued care provision due to: death of the care recipient (*n* = 1); institutionalization (*n* = 2); or transference of care responsibilities to another informal carer (*n* = 1). Among those who continued caregiving, the number of hours spent caring increased at post-test (Mdn 36, IQR 66 at T_0_ vs. Mdn 40, IQR 63 at T_1_), followed by a decrease at follow-up (Mdn 30, IQR 80).

Scores on attitudes towards online interventions decreased significantly over the three assessment waves in the control arm (χ^2^_(2, *N* = 20)_ = 7.882, *p* = .019; M_T0_ 21.3, M_T1_ 19.5, M_T2_ 19.9), but not in the intervention arm (χ^2^_(2, *N* = 11)_ = 1.429, *p* = .490; M_T0_ 22.5, M_T1_ 22.2, M_T2_ 22.2).

### Feasibility and engagement

The study participation rate was fair (78.1%), and higher for referred participants as compared to volunteers (81% vs. 76%) (Table 3). Nonparticipation was either due to the dead of the care recipient or to unknown reasons. The retention rate was also fair (73.8%) and higher for referred participants (100%) than for volunteers (58%). Accounting for carers showing interest in participating (27 referred, 37 volunteers; Fig. 1), 59.2% of referred participants and 40.5% of volunteers are study completers. The retention rate was lower for the intervention arm (52.4%) as compared to the control arm (95.2%). Reasons for dropout are not quantified but are explored in the interviews.

Engagement data for the intervention group shows that 3 carers have never registered into iSupport. Technical aid to register was requested by two carers. Participants visited a median of 13 lessons; however, 3 carers visited less than 5. Most study non-completers from this arm (*N* = 10) had an attendance rate below 20%, but two showed above the average attendance (56.5, 91.3%). Participants logged in into the program on a median of 6 times until post-test (range 1–20); however, they may remain logged. Over a third of participants still visited iSupport at post-test. The median time until discontinuation was of 2 weeks. Mood rating and printouts were used by most carers but not often. Two one-time-only visitors printed the lessons, suggesting offline use of iSupport.

**Table 3:** Feasibility and engagement data

Feasibility		Engagement (***N*** = 21)	
Participation rate, n (%)		Non-use attrition (T_1_), n (%)	3 (14.3)
All participants	50 (78.1)	Intervention dropout (< 5 lessons), n (%)	3 (14.3)
Referred participants	22 (81.0)	Usage at T_1_ ,Yes, n (%) ^#^	7 (38.9)
Volunteers	28 (76.0)	Weeks until discontinuation, Mdn (IQR) ^*^	2 (5)
Non-eligibility rate, n (%)	7 (14.0)	Logins into iSupport (T_0_-T_1_), Mdn (IQR) ^#^	6 (11.3)
Study dropout at T_1_, n (%)		Lessons visited, Mdn (IQR) ^#^	13 (16.5)
All participants	7 (16.7)	Average attendance rate, M (SD) ^#^	53.7 (34.4)
Intervention arm	6 (28.6)	Visits per module, n (%) ^#^	
Control arm	1 (4.8)	Module 1	16 (88.9)
Study dropout at T_2_, n (%)		Module 2	15 (83.3)
All participants	4 (9.5)	Module 3	13 (72.2)
Intervention arm	4 (19.0)	Module 4	14 (77.8)
Control arm	0	Module 5	11 (61.1)
Retention rate (from T_0_ to T_2_), n (%)		Mood rating function	
All participants	31 (73.8)	Used, Yes, n (%) ^#^	17 (94.4)
Intervention arm	11 (52.4)	Visits, Mdn (IQR) ^#^	1 (.3)
Control arm	20 (95.2)	Printout function, Used, n (%) ^#^	17 (94.4)

*Abbreviations*: *N* number of participants, *M* mean, *Mdn* median, *IQR* interquartile range, *T*_*1*_ 3 months after baseline, *T*_*2*_ 6 months after baseline. ^*^N = 11 (excluding participants not registered, and those still using iSupport at T_1_); ^#^*N* = 18 (excluding not-registered participants)

### Group-time effect: per-protocol and intention-to-treat

Following a per-protocol approach (Table 4), significant group-by-time interaction effects were found for anxiety symptoms (Wald *χ*^2^ = 6.17*, p* = .046), as well as for quality-of-life, environment domain (Wald *χ*^2^ = 7.06*, p* = .029). A decreasing trend of anxiety scores in all three assessment moments was found for the intervention arm, while for the control arm those scores increased slightly from baseline to post-test and decreased by follow-up. Environment quality of life scores slightly improved in iSupport group; however, these have decreased for the control group. For intention-to-treat analyses adjusted for age, however, group-by-time interaction effects are no longer statistically significant (Supplementary Table).

**Table 4:** Descriptive statistics for outcome measures at T_0_, T_1_ and T_2_ per group, and generalized estimating equations (GEE) model parameters for group-by-time interaction according to the per-protocol analyses (*N* = 31)

	iSupport (***N*** = 11)			Control (***N*** = 20)			Group-time effect		
Outcomes	T_**0**_	T_**1**_	T_**2**_	T_**0**_	T_**1**_	T_**2**_	B ^**†**^ (95% CI)	χ^**2**^	p^*^
Caregiver burden (ZBI)	37.8 (13.1)	37 (9.8)	33.6 (14.8)	37.1 (11.2)	34.7 (11.4)	32.9 (11.8)	−.000 (−.15, .15) .05 (−.15, .25)	.45	.800
Anxiety symptoms (HADS-A)	10.1 (4.6)	8.3 (4.9)	7.9 (5.3)	8 (3.4)	8.4 (2.9)	7.2 (3.5)	.14 (−.08, .36) −.11 (−.39, .16)	6.17	**.046**
Depression symptoms (HADS-D)	6.6 (5.2)	7.4 (3.5)	6.7 (3.1)	6.1 (4.0)	8.5 (2.8)	7.3 (2.7)	.09 (−.25, .44) −.07 (−.31, .17)	1.31	.347
Positive aspects of caregiving (PAC)	35.8 (6.1)	36.6 (5.6)	38.7 (6.4)	39.6 (8.4)	40.5 (9.2)	39.5 (10.5)	−.08 (−.19, .02) −.08 (−.16, −.001)	4.16	.125
General Self-efficacy (GSE)	28.5 (4.9)	30.1 (3.0)	31 (4.0)	31.3 (4.7)	32 (5.5)	30.9 (4.9)	−.10 (−.20, .01) −.07 (−.14, .004)	4.43	.109
QoL physical (WHOQOL-BREF)	26.5 (4.4)	26.2 (5.1)	27.6 (4.5)	27 (4.4)	25.9 (4.5)	26.1 (4.5)	−.07 (−.19, .04) −.04 (−.17, .08)	1.97	.374
QoL psychological (WHOQOL-BREF)	22.7 (4.6)	22.6 (4.0)	23 (4.0)	22.9 (3.8)	21.3 (3.1)	21.9 (3.2)	−.06 (−.15, .03) .01 (−.07, .09)	3	.223
QoL social relationships (WHOQOL-BREF)	10.4 (2.8)	10.3 (2.3)	10.6 (2.9)	10.1 (2.6)	9.4 (2.1)	9.5 (2.5)	−.09 (−.23, .05) −.02 (−.16, .12)	2.41	.299
QoL environment (WHOQOL-BREF)	29.3 (4.0)	30.1 (4.4)	30.6 (4.5)	29.3 (4.2)	28.4 (3.8)	27.7 (3.8)	−.10 (−.18, −.02) −.04 (−.10, .02)	7.06	**.029**
QoL general (WHOQOL-BREF)	7.6 (1.3)	7.6 (1.6)	7.8 (1.2)	6.8 (1.5)	6.9 (1.0)	7 (1.1)	−.007 (−.11, .10) −.01 (−.13, .10)	.06	.973

*Abbreviations*: *N* number of participants, *M* mean, *SD* standard deviation, *CI* confidence interval, *T*_*0*_ baseline assessment, *T*_*1*_ 3 months assessment, *T*_*2*_ 6 months assessment, *ZBI* Zarit Burden Interview, *HADS-A* Hospital Anxiety and Depression Scale (anxiety subscale), *HADS-D* Hospital Anxiety and Depression Scale (depression subscale), *PAC* Positive Aspects of Caregiving, *GSE* Generalized Self-efficacy Scale

^*^P-values for type III GEE model effects tested using the Wald Chi-Square test. Values in bold represent the statistically significant differences at *p* < .05

Betas are presented as unstandardized coefficients with the respective 95% confidence intervals.

^†^ Unstandardized coefficients and 95% confidence intervals values under group-time effect corresponding to group 1 * time 1 and group 1 * time 2 (upper and lower values, respectively)

### Characterization of interviewees

Twelve carers allocated to the intervention arm were interviewed, including 4 study non-completers. Non-completers are participants who did not fill all the assessment waves; however, those adhered differently to the intervention: 2 did not register on iSupport, thus not only are they classified as non-completers, but they are also counted towards the non-use attrition reported in Table 3 (14.3%, *n* = 3); 1 visited 13 lessons and discontinued after 2 weeks; and 1 visited 21 lessons and still used iSupport at post-test. Nine female and 3 male carers were interviewed, aged 49.3 years on average (SD 9.1). Three participants had 12 or less years of schooling while nine had at least a degree. Most interviewees were children of the person with dementia (*N* = 10), except for 2 spouses. Carers appraised the care recipients’ degree of dependence as severe/total (*N* = 8), or mild/moderate (*N* = 4). Most carers lived with the care recipient (*N* = 7), cared for a median of 30 h./week (IQR 46) and 4 years (IQR 3). Both carers providing less intensive (< 20 h./week; *N* = 5) and more intensive care (> 20 h./week; *N* = 7) were represented; but all, except one, were providing long term care (1.5–10 years). Either carers being the only source of care (*N* = 4) or sharing caregiving (*N* = 8) were represented. The interviews lasted 57.8 min on average and the transcribed verbalizations were analysed.

### Thematic content analysis

The emergent themes from the analysis are described below. Trends are expressed by number of references for an issue (R), participants contributing to the issue (N), and/or quotations from carers (IC), which were translated into English.

1. Motivations to participate (R = 26)

Motivations included: a) getting information about the disease and strategies to care for the person with dementia (*N* = 9; *R* = 11); b) reaching out emotional support (*N* = 4; R = 6) – *“I was curious to see how could this program help me, at a psychological level… a person needs emotional support”* (IC9); c) not having other forms of support (*N* = 4; *R* = 4) – *“In Portugal…there are no many responses, are there? Besides the support line and ‘Memory Cafe’, I do not think we have other kinds of support”* (IC5); and d) the unique advantages of online interventions (*N* = 3; *R* = 5) – “*I cannot leave home anymore, leaving my husband alone. If it were not for online support, I wouldn’t have any support!”* (IC3).

2) Advantages and drawbacks of online training and support (*R* = 50)

The accessibility and the immediacy of online interventions were seen as the main advantage (*N* = 10; *R* = 18) – “A*ccessing it from my computer or smartphone, having it available all the time and doing it at my rhythm, is an added value”* (IC5). Carers reporting to feel uncomfortable in sharing emotions in a group, see online interventions as an alternative way of getting support (*N* = 4; *R* = 7) – *“I see advantage on not needing to share…I’m aware that I could enrol in support groups, but I never felt comfortable to go. I keep imagining that kind of ‘round table’ where I would feel pressured to share”* (IC12).

Drawbacks of online interventions comprised: a) the exclusion of persons who are less educated or digitally illiterate (*N* = 9; *R* = 17); and b) the lack of interactivity or non-verbal aspects characterizing in-person communication (*N* = 3; *R* = 3) – *“online options will never replace in-person training. Why? Because the online can never have the emotional aspect of an in-person conversation. I speak not only with my voice but with my gestures”* (IC1).

3) Usage of iSupport (*R* = 95)

Most carers used the program through a smartphone, and all reported different usage patterns. However, none of the interviewees have established a schedule to visit the program – *“I never used iSupport as an obligation, like an online course. I knew that I had it there for me to use every time I felt I needed it”* (IC1). Interviewees reported either: a) having followed the lessons sequentially (*N* = 5; *R* = 8); or b) having chosen the lessons according to their needs (*N* = 7; *R* = 9; 2 participants did both) – *“I had different reasons to visit the program. Sometimes I needed to be comforted, other times I wanted to clarify a doubt about practical issues (…) so I choose the lessons according to those needs*” (IC1). Interviewees were fairly divided among those who used (*N* = 6) and not used (*N* = 6) iSupport as much as they would like to. Lack of time was the reason evoked for not using iSupport as desired – *“I used it less than I would like to. I would like to have finished all the lessons, but my days would need to have 48 hours”* (IC7). In exploring the motives for non-adherence among the interviewees who did not register into iSupport (*n* = 2), lack of time was mentioned by both, including due to increased caregiving demands during the lockdown.

The use of iSupport was perceived by some carers (*N* = 8; *R* = 16) as being negatively affected by increased caregiving demands during the COVID-19 outbreak - *“With the lockdown, the responsibility of caring for my mom is entirely on me now, with no help from the day center or my sister… plus home schooling, teleworking. This is a good resource, useful, but I’m so tired that sometimes I don’t have the energy to use it”* (IC11). There were also reports of a positive influence, due to having more free time during lockdown (*N* = 4; *R* = 4).

Most carers (*N* = 8) reported not having used any psychosocial service concomitantly with iSupport. The carers who did it (*N* = 4) participated in psychoeducational or support groups. None of the study non-completers received concomitant support.

4) Satisfaction with iSupport contents, design, and functionalities (R = 137)

In overall, iSupport lessons were described as comprehensive and the themes matched the carers’ most prominent needs (*N* = 9; *R* = 17) - *“the program approaches the main issues that we face as carers in a daily basis, so it is a good resource for us”* (IC1). However, missing themes were identified (N = 9; *R* = 34), including: fall prevention/management; anticipatory grief; self-understanding/self-compassion; relevant legislation; and challenges associated the most with later disease stages (e.g., tube feeding) – *“the problems that people have at a later stage of the disease are not addressed in this program. I think it is directed for carers of someone in initial stages”* (IC2). All participants appreciated being allowed to choose the themes/lessons.

Interviewees considered the language in iSupport *“easy to understand”* (IC7), *“accessible”* (IC5), and *“free of medical terms”* [jargon] (IC9). Most (*N* = 9; *R* = 18) enjoyed the presentation of contents in text but would feel more engaged/use iSupport more with additional multimedia resources – *“A person needs to sit down and be more focused to read the lessons. I would use it more if I could just listen to the lessons. I could do other things, like making dinner, while listening”* (IC3). As most carers are smartphones users, recommendations to launch an iSupport *app* were made (*N* = 3; R = 4) – *“the program would be more helpful as an app… I would use it more…the simple icon on the screen is a reminder to use it”* (IC6).

Carers perceived iSupport’s interface as “*intuitive”* (IC1) and “*easy to use”* (IC5), but user experience in mobile devices was less interesting due to visualization issues (*N* = 3; *R* = 4). Participants valued the interactive features of iSupport (*N* = 4; *R* = 6); while would expect more personalization (*N* = 3; *R* = 4) – *“there is a part about intimacy… I care for my mom, so it doesn’t make sense to me (…) I would like to have information more… personalized to my situation”* (IC9). Most participants would like to find in iSupport a mean to interact with either professionals (*N* = 7; *R* = 9) or peers (*N* = 7; *R* = 7) – *“a chat would be good, to clarify any doubt with a professional, and I think that would encourage me to visit the program”* (IC7).

5) Perceived results of iSupport and program endorsement (*R* = 67)

Most carers, except one, perceived positive results from using iSupport. The carer not identifying positive results considered iSupport as unhelpful for supporting a person in the later stages of dementia.

Perceived positive results included: a) acquiring new knowledge about the disease or learning new strategies to manage everyday care (*N* = 7; *R* = 16) – *“All those tips, information, and those tables about eating and drinking or about going to the bathroom… I wouldn’t do that intuitively…it was a great help for everyday care”* (IC1); and b) experience positive processes and feelings (*N* = 7; *R* = 26) including self-understanding/self-compassion – *“for me it was important to accept the idea that I won’t’ be able to do everything and I’ll commit mistakes but I’m doing my best and I can’t live feeling guilty”* (IC7); feeling understood/not ‘feeling alone’ – “*The best result for me was that while I was reading, I was relating with those situations and thinking ‘if this is here, other people must be experiencing the same situations thus…I’m not alone’…that thought made me feel better”* (IC9); being more willing to take care for oneself – “*the program makes me focus not only on the disease he has…it makes me want to also take care of myself”* (IC3); and validation of their actions as carers - *“what happened a lot was: I had an idea to deal with some issue, and the program suggested something similar. That was a pretty good feeling, that kind of reinforcement. Sometimes I searched the program to kind of validate my ideas”* (IC5). No adverse results were reported.

Most interviewed carers, except one (caring for a very dependent person), would endorse the programme (R=17) – “*I fully support this initiative, there are not many for us*. *I have no doubts that I would recommend it, in fact I did it already”* (IC1).

6) Satisfaction with study procedures (*R* = 32)

None of the participants was unsatisfied with study procedures and no major inconvenient was reported. Weekly reminders to use iSupport and to fill assessment forms were the two main study procedures. Among study dropouts, no reason besides lack of time was evoked for not filling all three assessment waves. The assessment forms were considered adequate with respect to time of completion and questions asked, including from the perspective of study dropouts. The reminders were in overall favoured by participants (*N* = 10; *R* = 16) also as a mean to increase adherence (*N* = 3; *R* = 3) – *“The reminders were really helpful because is really easy for us to forget about taking care for ourselves…actually, I think these should be part of the program, that would be an incentive.”* (IC1). Two participants referred to feel pressured by the reminders. One participant suggested periodic videoconference meetings during the study to increase engagement.

## Discussion

This pilot study is to the best of our knowledge the first in Portugal to evaluate the feasibility of an online training and support program for dementia carers. Internationally, this is among the first studies providing insights about a culturally adapted version of the WHO’s ‘iSupport’ program. Insights are offered at two levels: 1) for implementing an optimized full-scale effectiveness trial; and 2) for understanding the acceptability and potential results of iSupport-Portugal, as well as for improving its features before a full release.

### Insights for a full-scale effectiveness trial

The participation as well as the non-eligibility rates (78.1% and 14%) were fair. Inclusion criteria based on psychological needs had no role in excluding participants: all candidate participants experienced clinically relevant levels of burden, with or without associated anxiety and/or depression, confirming high psychological needs in Portuguese dementia carers [28, 29]. The retention rate of randomized participants was also fair (73.8%), however lower for the experimental as compared to the control group. Such differences might relate with participants not being blind to their allocation condition, as the control group might stay retained to access iSupport at the end of the study. The overall retention rate in this pilot is aligned to previous research [21] and is higher as compared to the Indian iSupport’s trial (36.42%) [35]. This suggests the feasibility of a full-scale RCT in Portugal.

The initial study protocol foresaw a referral-based recruitment [36]. With the COVID-19 outbreak and the suspension of psychosocial services, we also conducted advertising-based recruitment. While this was a contingency measure, we took the chance to examine the advantages and drawbacks of each recruitment strategy. Volunteers were recruited at a higher speed and number as compared to referred carers. However, both the participation and retention rates were higher for referred participants, suggesting that this might be a more efficient recruitment strategy. Advertising-based recruitment carries a chance of volunteer bias and is often avoided. However, participants recruited by referral are also more likely to have been acquainted with support services. Mixing and controlling for both recruitment methods may be an interesting approach in a future trial. In this pilot, due to sample size limits, we compared recruitment strategies for feasibility parameters but not for intervention effects.

A not-neglectable number of participants randomized to iSupport never registered into the program or visited a residual number of lessons (28.6%, *N* = 6). In India, 69% of carers completed less than 5 lessons [35], suggesting a higher adhesion among Portuguese carers participating on this pilot. Weekly email reminders implemented to prevent attrition may have had a role in prompting participation, as suggested by interviews. The drawback of reminders is that when not embedded as an intervention feature, those may lead to an overestimation of ‘real-life’ engagement data.

Study non-completers were significantly younger than completers, in line with previous research [35, 47]. Importantly, study dropout was more frequent among carers scoring higher on anxiety, suggesting that carers who are more in need stay less retained in the intervention. This drawback is not unique for iSupport, as previous research reported less use and retention in interventions by persons in worse mental health [29, 48]. Research has also shown that participants with worse *à priori* attitudes towards interventions are less likely to dropout and benefit the most from interventions [49]. We have added to the protocol a measure on attitudes towards online interventions [31] and compared completers and non-completers, who were not different; however, whether attitudes relate to outcomes are a matter of study for a full-size trial.

This pilot had limitations that must be addressed in a forthcoming study. First, as we initially foresaw a referral-based recruitment only, the protocol did not include any validated measure on dementia screening/functional decline (e.g., AD8). Also, access to psychosocial services during the trial must be controlled for a potential role/benefit of other interventions. Half of interviewed study-completers benefited from psychosocial support during the study; however, we miss this information for the control group. Indeed, participants in the control group were not interviewed, thus there are open questions on e.g., the extent to which the e-book was used and considered beneficial; or why attitudes towards online interventions have become more negative over time in this group. In a future RCT, representatives of the control group must be interviewed, and qualitative explorations with both groups must focus more on the outcomes of the intervention (iSupport or education only e-book) for the carer and the care recipient.

This pilot also confirmed our previous assumption [36] that participants would probably be highly educated, since education is an important determinant of internet use [50]. A forthcoming full-scale study will face generalization issues of the results to the entire population of dementia carers.

In a future study, collecting fine-grained usage data would be instrumental accounting for the self-guided nature of iSupport, including on the amount of time spent on the program and engagement with interactive exercises. Exercises are instrumental for skills training; however, we were not able to retrieve data on whether carers performed such exercises. One must also consider including other outcomes of interest. Since interviewed carers perceived knowledge gains and experienced positive processes and feelings by using iSupport, including as outcomes of a future study, knowledge, feelings of guilt and loneliness, feelings of validation (as a carer), and self-care behaviours, may yield relevant findings. Future research must also offer insights on whether an online intervention as iSupport may affect care recipient’s outcomes or outcomes related to the carer’s relationship with the person with dementia. A recent systematic review [19] concludes that such outcomes are barely studied in RCTs of training and support programs for informal dementia carers.

Most internet intervention trials based of cognitive-behavioural therapy techniques replicate the timings of face-to-face interventions, collecting repeated measurements 3 and 6 months after baseline. Out pilot was not an exception. However, carers discontinued iSupport in about 2 weeks, while visited on average half of lessons, suggesting an intense program use, in line with previous research [51]. The assessment timings might worth rethinking, as post-test and follow-up measurements may be more sensitive if collected shortly after baseline. The control group showing more negative attitudes towards online interventions over time, may reflect unsatisfaction with the time until getting access to iSupport (only after all assessments).

### Insights on iSupport-Portugal

Following a per-protocol approach, intervention effects were suggested for anxiety symptoms and environmental quality of life. Anxiety scores decreased in the intervention arm over time, while slightly increased in the control group from baseline to post-test. The subjective appraisal of environmental quality of life slightly improved in the intervention arm while decreased on the control group. As the WHOQOL-BREF environment subscale addresses the satisfaction with access to information or health services, the intervention arm may perceive more access than the control arm who received an e-book only. Following an intention-to-treat analysis adjusted for age (Supplementary Table), however, group-by-time interaction effects are no longer statistically significant, a finding that must be seen in the context of a pilot study with a small sample. For pilot studies, it was previously stressed that the significance level can be increased for hypothesis testing and to preliminarily explore the effects of an intervention [52]. As in the Indian trial [35], no intervention effects were found for burden of care, depression or self-efficacy, irrespective of the approach to the data (per-protocol or intention-to-treat). The insights provided by the per-protocol approach are relevant when the objective of the study is explanatory [53], and offer information on whether the intervention produces effects among those who really receive it. However, this approach can lead to biased results and overestimate the benefit of the intervention, especially if it accounts for the ‘best-case scenario’ with respect to adherence. In the context of iSupport (an online self-guided intervention allowing the carer to select the own plan of lessons), defining what means ‘receiving the intervention’ is not straightforward. For this pilot, it meant visiting a minimum number of lessons (5 lessons, according to WHO’s recommendations in the original iSupport). Thus, there is variability among completers with respect to the use of the program, what may affect the results of per-protocol analyses. Intention-to-treat analyses were carried to ‘bracket’ the likely effects under different conditions. These analyses however, required multiple imputation of missing data, a procedure debated for its validity when used in small samples [54]. A full-powered effectiveness study is needed for robust conclusions, as a pilot study has neither the intention nor is it formally powered to assess effect. The forthcoming RCT must also explore whether iSupport produces different effects according to the characteristics of carers, care recipients, and of the context of care.

Further implementation research on iSupport-Portugal is welcome to explore the different possibilities in implementing the program (e.g., design choices, delivery format, professional/peer interaction) and how these may influence the effects of the program. Interview data revealed a good acceptance of iSupport. Carers would however like to find more personalized features, as well as more diverse, ‘easy to consume’ and engaging formats for content presentation. Also, while iSupport was developed for computer or tablet screens, most carers used smartphones and reported interface issues affecting the experience. Converting iSupport into an *app*, which is more visible and available than a web platform, and embedding the weekly reminders as an iSupport feature, were suggested as means to promote the uptake of iSupport.

Contents of iSupport were described as comprehensive and useful. Lessons were however deemed inadequate for carers of persons in later disease stages. Late access to psychosocial interventions, is commonly described among dementia carers [30]. We registered the death or institutionalization of the person in care among 9.7% of candidate participants during the pilot, which might suggest a severe health status of the person in care when help was sought-after by carers. While iSupport should ideally be accessed at early stages, a pragmatic approach may require adapting the program contents to later ones.

Interview data suggest that carers would favour the online interaction with professionals or peers, even those reluctant with such interaction in-person. Professionals involvement in self-help interventions was reported to improve intervention retention and outcomes [12]. Embedding these features into iSupport is a case of study for Portugal, although a drawback would be a significant raise in service costs, thus a health economics perspective is needed in further iSupport studies. The high retention of referred participants in this study also suggests that, in the future, it is instrumental to raise awareness of the program among professionals. These professionals may act as ‘prescribers’ of iSupport and recommend specific modules within the program according to carers’ needs.

When fully releasing iSupport, and to follow-up on usage data, the pilot suggests issues to consider. First, cases where the printouts were used massively in a one-time-only visit to iSupport, suggest that offline use may occur, and online use may be underestimated. Second, most carers skipped satisfaction ratings requested by the program at the end of each lesson. Prompting such assessments would be helpful to get substantial satisfaction data in the future.

## Conclusions

Offering accessible, acceptable, and effective interventions for informal dementia carers is a strategic priority in the Global Action Plan on the Public Health Response to Dementia [55]. This study suggests that a European-Portuguese version of the WHO’s iSupport program has good acceptability and promising preliminary results on carers mental health, knowledge, and well-being. However, a full-scale RCT is needed to determine iSupport’s effectiveness and this pilot suggests its feasibility together with measures to optimize the study protocol. Improvements on iSupport’s contents and interface are also suggested. Accounting for the high prevalence of dementia and high rate of informal home care in Portugal, iSupport may be a relevant adjunct support for Portuguese carers.

## Supplementary Information

**Additional file 1.** CONSORT 2010 checklist of information to include when reporting a pilot or feasibility trial*.

**Additional file 2: Supplementary Table**. Generalized estimating equations (GEE) model parameters for group-by-time interaction according to the Intention-to-treat analyses (*N* = 42, 21 per group).

## Data Availability

Relevant raw data will be freely available to any researcher wishing to use them for non-commercial purposes, without breaching participant confidentiality, and upon request to the authors.

## Footnotes

1 The results of the Dutch trial were not yet published at the time of submission of this manuscript.

2 All potential participants filling the online form and presenting high scores for burden and/or severe symptoms of anxiety or depression, visualized an automated message recommending the consultation of local health services and/or reaching out for the help of a trusty person. The contacts of the research team were displayed as a mean to ask for guidance support-seeking.
