# Supplementary material for: Feasibility of an online training and support program for dementia carers: results from a mixed-methods pilot randomized controlled trial
Source: BMC Geriatr. 2022 Mar 1;22:173. doi: 10.1186/s12877-022-02831-z (PMC8887647; doi:10.1186/s12877-022-02831-z)
Supplement: Supplementary file 2 — Additional file 2: Supplementary Table. Generalized estimating equations (GEE) model parameters for group-by-time interaction according to the Intention-to-treat analyses (N = 42, 21 per group). [file 12877_2022_2831_MOESM2_ESM.docx]

**Supplementary Table.** Generalized estimating equations (GEE) model parameters for group-by-time interaction according to the Intention-to-treat analyses (N = 42, 21 per group).

|  | **Group-time effect** | |
| --- | --- | --- |
| **Outcomes** | **B (95% CI)** | ***p*** |
| Caregiver burden (ZBI) | 1.1 (-1.14, 3.33) | .338 |
| Anxiety symptoms (HADS-A) | -.34 (-1.27, .59) | .477 |
| Depression symptoms (HADS-D) | -.61 (-1.7, .49) | .275 |
| Positive aspects of caregiving (PAC) | 1.22 (-.78, 3.2) | .231 |
| General Self-efficacy (GSE) | .29 (-1.03, 1.62) | .662 |
| QoL physical (WHOQOL-BREF) | .32 (-1.03, 1.67) | .645 |
| QoL psychological (WHOQOL-BREF) | .52 (-.47, 1.50) | .306 |
| QoL social relationships (WHOQOL-BREF) | .44 (-.37, 1.25) | .284 |
| QoL environment (WHOQOL-BREF) | .54 (-.80, 1.89) | .432 |
| QoL general (WHOQOL-BREF) | -.16 (-.58, .27) | .472 |

**Abbreviations:** n – number of participants; CI – confidence interval; ZBI – Zarit Burden Interview; HADS-A – Hospital Anxiety and Depression Scale (anxiety subscale); HADS-D – Hospital Anxiety and Depression Scale (depression subscale); PAC - Positive Aspects of Caregiving; GSE - Generalized Self-efficacy Scale.

Betas are presented as unstandardized coefficients adjusted for age with the respective 95% confidence interval.

**^†^** Unstandardized coefficients and 95% confidence intervals values under group-time effect corresponding to group 1 * time (group 2 * time as reference)
